# Chinese Herbal Medicine Xingnaojing Injection for Acute Ischemic Stroke: An Overview of Systematic Reviews and Meta-Analyses

**DOI:** 10.3389/fphar.2021.659408

**Published:** 2021-05-18

**Authors:** Zi-Yu Tian, Lu-Da Feng, Yao Xie, De-Hao Xu, Chong-Yang Zhang, Ling-Bo Kong, Rong-Rong An, Li-Fang Ma, Nicola Robinson, Ying Gao, Jian-Ping Liu

**Affiliations:** ^1^ Dongzhimen Hospital, Beijing University of Chinese Medicine, Beijing, China; ^2^ Centre for Evidence Based Chinese Medicine, Beijing University of Chinese Medicine, Beijing, China; ^3^ Institute for Brain Disorders, Beijing University of Chinese Medicine, Beijing, China; ^4^ Department of Neurology, Affiliated Hospital of Hunan Academy of Traditional Chinese Medicine, Changsha, China; ^5^ Beijing Tongzhou Xinhua Hospital, Beijing, China; ^6^ School of Health and Social Care, London South Bank University, London, United Kingdom

**Keywords:** Chinese herbal medicine, Xingnaojing injection, acute ischemic stroke, overview, systematic reviews, meta-analyses

## Abstract

**Background:** Xingnaojing injection (XNJ) is the only Chinese herbal injection approved for both intracerebral hemorrhage and ischemic stroke (IS) first-aid on ambulances in China; many systematic reviews (SRs) and meta-analyses (MAs) of XNJ on stroke have been published. The purpose of this research was to evaluate and summarize the current evidence on XNJ for IS.

**Methods:** A comprehensive search was conducted for SRs and MAs of XNJ on IS in seven databases up to January 1, 2021. Two authors independently identified SRs and MAs, extracted data, assessed the quality of the included SRs and MAs using the Assessment of Multiple Systematic Reviews 2 (AMSTAR 2), and assessed quality of evidence using the Grading of Recommendations, Assessment, Development, and Evaluation (GRADE).

**Results:** A total of 10 SRs met the inclusion criteria. The quality of included SRs using AMSTAR 2 was critically low as the critical items were poorly reported. Only 10% of SRs reported 50% of the 16 items, while the remaining 90% SRs reported just less than half of the items on AMSTAR 2. For GRADE, 7 (35%) of outcomes had low-quality evidence, 10 (50%) with very low, and 3 (15%) with moderate quality evidence. Very low to low quality of evidence indicated XNJ plus conventional therapy (CT) alleviated the neurological deficits of acute IS. Moderate-quality evidence showed XNJ plus CT reduced mortality when compared to Danshen injection plus CT, and very low-quality evidence showed XNJ plus CT could not improve the degree of coma, while low-quality evidence indicated the opposite. Mild adverse events in the CT group were less than those in the XNJ plus CT group, and there were no serious adverse events, but there was no statistical difference between the two groups. The included 10 SRs indicated that XNJ was used for acute IS, but only four SRs (40%) reported the course of disease.

**Conclusion:** XNJ appears to be effective and safe for acute IS in improving the neurological deficits, but the evidence is not robust enough. However, whether administering XNJ immediately after or within 24 h of IS is best remains unknown due to the lack of data. Well-designed large-scale randomized controlled trials with measurable outcomes are required in future studies.

## Introduction

As the second leading cause of deaths worldwide, stroke is also problematic because it results in high morbidity, disability, and recurrence (GBD 2016 Stroke Collaborators, 2019; GBD 2016 Causes of Death Collaborators, 2017). In addition, there is a large economic burden due to the various life-influencing handicaps experienced by stroke survivors who need extensive and individual treatment and healthcare (Rajsic et al., 2019). Ischemic stroke (IS) is the main subtype of stroke with the proportion more than 80% (Benjamin et al., 2019).

Vascular recanalization strategies including intravenous thrombolysis and endovascular treatment are recommended to timely accelerate reperfusion (Powers et al., 2019). Unfortunately, although substantial advances in treatment at the acute stage of IS have emerged in recent years, only a minority of patients can clinically benefit from vascular recanalization treatment (Liu et al., 2011; Demaerschalk et al., 2016). The unavoidable reasons for this strict time-window restrictions include, increased risk of hemorrhagic transformation, imaging dependence and technical surgical operation requirement (Yaghi et al., 2017; Powers et al., 2019). It will be hard to reverse the functional status of IS survivors once they miss the crucial 24 h immediately after stroke onset. There are many potential neuroprotective agents that can be explored, but randomized controlled trials (RCTs) are required to prove their effectiveness.

Xingnaojing injection (XNJ) is one of the China Food and Drug Administration’s approved neuroprotective agents for the treatment of acute stroke, and it is widely used in China (Wu et al., 2007). It is the only Chinese herbal injection approved for both intracerebral hemorrhage and ischemic stroke as a first-aid option on ambulances in China, but whether initiating treatment of XNJ for acute IS immediately after onset remains unknown. XNJ is extracted from a classical, well-known Chinese patent medicine that has been used clinically for more than 200 years. XNJ contains Moschus, *Borneolum syntheticum*, *Gardenia jasminoides* J.Ellis, and *Curcuma aromatica* Salisb. as well as appropriate amount of other ingredients. The theory of traditional Chinese medicine (TCM) believes that stroke is caused by phlegm-fire and other pathogenic factors that can affect the mind, reverse qi and blood, and result in blood stasis and block in the brain. Moschus can awake the brain and activate blood circulation, borneolum can also awake the brain and clear heat, while *Gardenia* has the effect of clearing heat and detoxification, and the *Curcuma* has the effect of promoting qi and blood circulation, so the ingredients together have the function of clearing heat and detoxification, cooling and activating blood circulation, and awakening the brain (Chinese Pharmacopoeia Commission, 2020). Basic research has shown the benefits of XNJ in treating IS, suggesting that XNJ is effective in alleviating inflammatory reactions, such as lowering TNF-a, IL-6, and IL-1b, in addition to improving the body’s antioxidant function (Ma et al., 2018). It is generally recognized that systematic reviews and meta-analyses are the cornerstones of evidence-based health care (Pieper et al., 2014), and such SRs on the use of XNJ for IS had been published. Previous systematic reviews (SRs) and meta-analyses (MAs) indicated that there was insufficient evidence to confirm the efficacy and safety of XNJ in treating IS (Li, 2006). The latest systematic review and meta-analyses suggested that there was significant benefit of XNJ in alleviating neurological impairment (Ma et al., 2017). In addition to the conflicts existing between different SRs and MAs, the exact time-window for initiating XNJ and duration of XNJ use for IS treatment were not documented in some studies (Li, 2006; Ma et al., 2013). Moreover, the primary outcomes of these studies were either a composite endpoint (cure rate, obvious effective rate, and effective rate) or the improvement of neurological impairment, which lacks robust support strength. As SRs and MAs with low quality may mislead clinical decision-making, a systematic overview that evaluates all systematic reviews can identify the quality of the methodology and the evidence from important outcomes using the GRADE approach. It has been acknowledged that overviews are valuable for clinical decision-making as they avoid uncertainty caused by conflicting conclusions from different reviews, and facilitate the discovery of potential evidence gaps (Smith et al., 2011; Yang et al., 2017). As the current evidence from different SRs and MAs about XNJ in treating IS has not previously been assessed systematically, we conducted this overview of systematic reviews to provide an overall evaluation of the quality of evidence and summarize the current evidence about XNJ for the treatment of IS.

## Methods

This overview was performed based on a predefined protocol drafted according to the Cochrane Handbook for conducting overviews of SRs and MAs of interventions (Higgins Jpt, 2019).

### Inclusion and Exclusion Criteria

Type of study: As RCTs are considered to provide high-quality evidence for assessing interventions, we included SRs of RCTs or quasi-RCTs assessing the effectiveness and safety of XNJ for IS.

Type of subjects: Subjects included had to be diagnosed as IS; there were no limitations on age, race, and gender.

Type of intervention: XNJ was used alone or combined with other therapies (like placebo, rehabilitation training, conventional therapy (CT, including thrombolysis, restoring blood supply to ischemic area, controlling cerebral edema, controlling hypertension, and reducing blood viscosity), and western medicine such as cerebral protection agents) in the treatment groups. The comparator interventions were CT, western medicine, placebo, rehabilitation training, or other herbal injection.

Type of outcome measures: Any effect-related outcomes were measured, such as mortality, disability, neurological deficit score, and adverse reactions, using activity of daily living assessment (like Barthel Index) and Glasgow Coma Scale (GCS).

Any duplicate publications were excluded. Conference abstracts were excluded if the relevant data were not supplied.

### Search Strategy

Seven electronic databases were searched without language limitation (from their inception to February 20, 2020). These included PubMed, EMBASE, Cochrane library, China National Knowledge Infrastructure (CNKI), China Science Technology Journal Database (VIP), Wanfang Database, and Sino-Med Database. We updated the search until January 1, 2021, before submission and included the latest published SRs and MAs that met the inclusion criteria. The searching strategies on PubMed and CNKI are listed in appendix 1 (Appendix 1).

### Study Selection and Data Extraction

Two reviewers (CYZ and DHX) independently identified SRs and MAs according to the predefined eligibility criteria and then independently extracted the basic information of the final included SRs and MAs according to the predesigned extraction table. Any disagreements were resolved by discussion or consultation with a third reviewer (YX). The basic information of SRs and MAs extracted were as follows: initials of the first author, publication year, number of included RCTs or q-RCTs, sample size, course and severity of IS, interventions in treatment and control groups, adverse events, outcomes, and main conclusions of included SRs and MAs.

### Quality Assessment

#### Methodological Quality by Assessment of Multiple Systematic Reviews 2

Two reviewers (CYZ and DHX) separately assessed the quality of included SRs and MAs using the Assessment of Multiple Systematic Reviews 2 (AMSTAR2) (Shea et al., 2017). Each of the 16 items was rated as “yes” (if the item was answered completely), “no” (the item was absent or not appropriate), or “partial yes” (some of the subitems incomplete). An overall rating (high, moderate, low, and critically low) was evaluated as follows: overall quality was assessed as high when there was no or just one noncritical weakness (item 2, item 4, item 7, item 9, item 11, item 13, and item 15 as critical items; others as noncritical items); moderate when there was just more than one noncritical weakness; low when there was just one critical flaw; and critically low when there was more than one critical flaw. Any discrepancies in the 16 items were resolved by discussion or judged by a third author (YX).

### Evidence Quality by Grading of Recommendations, Assessment, Development, and Evaluation

The Grading of Recommendation, Assessment, Development, and Evaluation (GRADE) system was used to assess the quality of the key outcomes (Guyatt et al., 2011). Two reviewers (YX and RRA) separately assessed the quality of the included SRs and MAs with GRADE; any discrepancies were resolved by discussion or judged by a third author (ZYT).

Five rating down factors were as follows: risk of bias, inconsistencies, indirectness, inaccuracy, and publication bias (Guyatt et al., 2008).

## Results

### Study Selection

A total of 235 articles were identified from seven databases. After 114 duplications were removed, we reviewed 121 records by title and abstract. And 31 were reviewed by full text. There were no SRs and MAs included after update retrieval. Finally, 10 studies meeting the inclusion criteria were included in our study (Xu et al., 2005; Li, 2006; Wang et al., 2006; Lin et al., 2010; Li et al., 2013; Ma et al., 2013; Chen and Gu, 2017; Ma et al., 2017; Wang et al., 2017; Liao et al., 2019).

**FIGURE 1:**
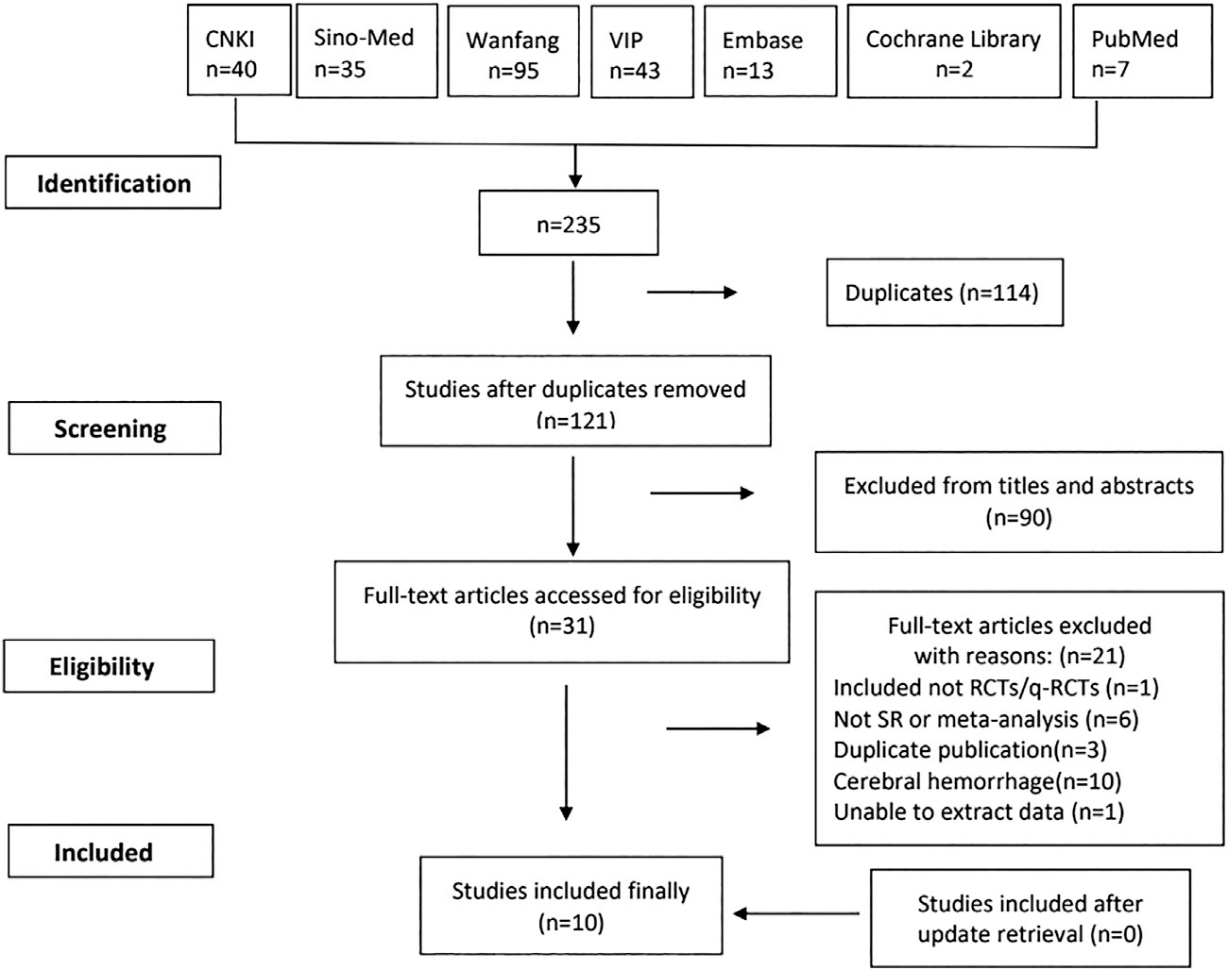
Flow diagram of literature selection.

The study flowchart is shown in Figure 1.

### Characteristics of Systematic Reviews

The characteristics of the included SRs and MAs are summarized in Table 1. A total of 10 studies were identified dating between 2006 and 2019 (Xu et al., 2005; Li, 2006; Wang et al., 2006; Lin et al., 2010; Li et al., 2013; Ma et al., 2013; Chen and Gu, 2017; Ma et al., 2017; Wang et al., 2017; Liao et al., 2019): only 1 SR was published in an English-language journal (Ma et al., 2017), and another nine were in Chinese-language journals (Xu et al., 2005; Li, 2006; Wang et al., 2006; Lin et al., 2010; Li et al., 2013; Ma et al., 2013; Chen and Gu, 2017; Wang et al., 2017; Liao et al., 2019). The first authors and the corresponding authors of the 10 SRs were from China (Xu et al., 2005; Li, 2006; Wang et al., 2006; Lin et al., 2010; Li et al., 2013; Ma et al., 2013; Chen and Gu, 2017; Ma et al., 2017; Wang et al., 2017; Liao et al., 2019). As for the included study type, 1 SR included RCTs or quasi-RCTs (Wang et al., 2006), and 9 SRs only included RCTs (Xu et al., 2005; Li, 2006; Lin et al., 2010; Li et al., 2013; Ma et al., 2013; Chen and Gu, 2017; Ma et al., 2017; Wang et al., 2017; Liao et al., 2019). The primary studies included in each SR ranged from 3 to 53, and the number of patients varied from 160 to 4,915. XNJ was used for acute IS in all SRs (Xu et al., 2005; Li, 2006; Wang et al., 2006; Lin et al., 2010; Li et al., 2013; Ma et al., 2013; Chen and Gu, 2017; Ma et al., 2017; Wang et al., 2017; Liao et al., 2019), and administration of XNJ in the patients with acute stroke was within 14 days from stroke onset in 2 SRs (Wang et al., 2006; Wang et al., 2017), within 30 days in 2 SRs (Li, 2006; Ma et al., 2013), and was unclear for the remaining 6 SRs (Xu et al., 2005; Lin et al., 2010; Li et al., 2013; Chen and Gu, 2017; Ma et al., 2017; Liao et al., 2019). All included SRs did not mention the severity of acute IS (Xu et al., 2005; Li, 2006; Wang et al., 2006; Lin et al., 2010; Li et al., 2013; Ma et al., 2013; Chen and Gu, 2017; Ma et al., 2017; Wang et al., 2017; Liao et al., 2019). As for the types of intervention, XNJ was used as an additive therapy, and the treatment in the intervention group was XNJ plus CT (Xu et al., 2005; Li, 2006; Wang et al., 2006; Lin et al., 2010; Li et al., 2013; Ma et al., 2013; Chen and Gu, 2017; Ma et al., 2017; Wang et al., 2017; Liao et al., 2019). While in the control group, the treatment in 6 SRs were CT (Lin et al., 2010; Li et al., 2013; Chen and Gu, 2017; Ma et al., 2017; Wang et al., 2017; Liao et al., 2019), 2 SRs were CT plus other therapy (Xu et al., 2005; Li, 2006), 1 was CT or CT plus other therapy (Wang et al., 2006), and there was no limitation on treatment in the control group in 1 SR (Ma et al., 2013). All the included SRs concluded that there was very low to low-quality evidence on the effectiveness of XNJ in acute IS. There is still a need for well-designed trials with large sample sizes to further prove the effectiveness and safety of XNJ in acute IS (Xu et al., 2005; Li, 2006; Wang et al., 2006; Lin et al., 2010; Li et al., 2013; Ma et al., 2013; Chen and Gu, 2017; Ma et al., 2017; Wang et al., 2017; Liao et al., 2019).

**TABLE 1:** Characteristics of included SRs and MAs.

Included SRs	No. of primary studies (patients)	Course of stroke	Severity of stroke	Adverse events (No. of patients in treatment group/control group)		Study types	Intervention measures		Outcome(s)	Main conclusion
							Treatment group	Control group		
Li. (2006)	3/160	<30 d	Unclear	There were no adverse events in included RCTs	RCT		XNJ + CT	CT	Total effective rate	There is no enough evidence to prove the efficacy and safety of XNJ for acute IS. More high- quality trials are required
Ma et al. (2017)	53/4,915	Unclear	Unclear	Not mentioned	RCT		XNJ + CT	CT	Overall response rate/neurological deficit score/serum levels of MMPs/hemorheology/blood lipid amelioration/hemodynamic/clinical symptom improvement	XNJ might be beneficial for IS
Ma et al. (2013)	11/961	<30 d	Unclear	There were no adverse events in included RCTs		RCT	XNJ + CT	No limitation	Total effective rate/neurological deficit score	XNJ might be beneficial for improving neurological impairment of acute IS
Wang et al. (2017)	24/2,514	<14 d	Unclear	Slight skin rashes, nausea or vomiting, headache, dizziness, drop in blood pressure, gastrointestinal reactions (17/21)		RCT	XNJ + CT	CT	Total effective rate/neurological deficit score/hemorheological parameters	XNJ combined with CT had good therapeutic effect on acute IS, while its safety still needed to be further investigated
Li et al. (2013)	36/3,114	Unclear	Unclear	Slight skin rashes (2/0)		RCT	XNJ + CT	CT/other therapy + CT	Total effective rate/mortality/cure rate/neurological deficit score/hemorheological parameters	XNJ may decrease mortality and increase the total effective rate of IS. More high-quality trials are required
Lin et al. (2010)	34/3,233	Unclear	Unclear	There were no adverse events in included RCTs		RCT	XNJ + CT	CT	Total effective rate/neurological deficit score/GCS/hemorheological parameters	XNJ may be superior to basic treatment in improving the total effective rate and neurological impairment. More high- quality trials are required
Xu et al. (2005)	13/1,203	Unclear	Unclear	Slight skin rashes (2/0)		RCT	XNJ + CT	Other therapy + CT	Total effective rate/mortality/cure rate/neurological deficit score	XNJ may decrease mortality and increase the total effective rate of IS. More high-quality trials are required
Chen and Gu, (2017)	16/1,310	Unclear	Unclear	Not mentioned		RCT	XNJ + CT	CT	Total effective rate/neurological deficit score	XNJ combined with CT could improve the total effective rate and neurological impairment of IS patients
Wang et al. (2006)	9/932	<14 d	Unclear	Slight skin rashes, nausea (4/0)		RCT/q-RCT	XNJ + CT	CT/CT + other therapy	Effective rate/mortality/neurological deficit score/hemorheological parameters	More high- quality trials are required to prove the efficacy and safety of XNJ for acute IS
Liao et al. (2019)	4/523	Unclear	Unclear	Slight skin rashes, nausea or vomiting, headache, dizziness (8/3)		RCT	XNJ + CT	CT	GCS	XNJ can improve the consciousness of patients with consciousness disorder after stroke, and has fewer adverse reactions and better safety

SRs, systematic reviews; MAs, meta-analyses; RCT, randomized controlled trial; q-RCT, quasi-randomized controlled trial; MMPs, matrix metalloproteinases; NIHSS, National Institutes of Health Stroke Scale; CSS, Chinese Stroke Scale; GCS, Glasgow Coma Scale.

### Effectiveness of Xingnaojing Injection for Acute Ischemic Stroke

Outcomes in the included 10 SRs were classified into three categories, including clinical outcomes related to the effectiveness of XNJ for acute IS, surrogate outcomes, and composite outcomes. We also classified and summarized the different scales for each clinical outcome, and the results were summarized as follows.

#### Clinical Outcomes

##### Outcome 1: Neurological Function

All included SRs assessed neurological function assessed by National Institutes of Health Stroke Scale (NIHSS), Chinese Stroke Scale (CSS), or European Stroke Scale (ESS) (Xu et al., 2005; Li, 2006; Wang et al., 2006; Lin et al., 2010; Li et al., 2013; Ma et al., 2013; Chen and Gu, 2017; Ma et al., 2017; Wang et al., 2017; Liao et al., 2019), but 3 SRs pooled the data of the RCTs that used NIHSS or CSS without subgroup analysis (Lin et al., 2010; Ma et al., 2013; Wang et al., 2017).

##### Scale 1: National Institutes of Health Stroke Scale

###### Xingnaojing Injection + Conventional Therapy vs. Conventional Therapy

Two SRs assessed the neurologic deficit score using the NIHSS (Li et al., 2013; Ma et al., 2017); the results of these two SRs showed NIHSS of patients in the XNJ group was much lower. One had no heterogeneity (Li et al., 2013) (3 RCTs, MD = −1.10, 95% CI −1.63 to −0.56, *p* < 0.00001, *I* ^*2*^ = 0%), while the other had high heterogeneity (Ma et al., 2017) (12 RCTs, MD = −3.44, 95% CI −4.52 to −2.36, *p* < 0.00001, *I* ^*2*^ = 92%), but the researchers did not explore potential factors for high heterogeneity.

##### Scale 2: Chinese Stroke Scale

###### Xingnaojing Injection + Conventional Therapy vs. Conventional Therapy

Three SRs compared XNJ combined CT with CT by CSS (Li et al., 2013; Chen and Gu, 2017; Ma et al., 2017); all results indicated the CSS in the XNJ group was much lower than that in the control group, although two SRs with high heterogeneity (Chen and Gu, 2017; Ma et al., 2017) (18 RCTs, MD = −5.72, 95% CI −6.94 to −4.50, *p* < 0.00001, *I* ^*2*^ = 87%; 5 RCTs, MD = −5.55, 95% CI −7.62 to −3.48, *p* < 0.00,001, *I* ^*2*^ = 86%) and one SR just included 1 RCT (WMD = −7.10, 95% CI −9.55 to −4.65) (Li et al., 2013).

###### Xingnaojing Injection + Conventional Therapy vs. Other Chinese Herbal Injections + Conventional Therapy

The results of two SRs showed XNJ can decrease the CSS compared to Danshen injection (RCTs = 6, WMD = −5.57, 95% CI −6.43 to −4.71, *p* < 0.00001, *I* ^*2*^ = 79.3%; 3 RCTs, WMD = 3.78, 95% CI 2.30 to 5.26, *p* < 0.0,001) (Xu et al., 2005; Li et al., 2013). One SR indicated XNJ can decrease the CSS compared to Shuxuetong injection (1RCT, WMD = −1.05, 95% CI −16.66 to −4.34, *p* = 0.0008) and Xuesaitong injection (1 RCT, WMD = −5, 95% CI −7.61 to −2.39, *p* = 0.0002) (Li et al., 2013).

###### Xingnaojing Injection + Conventional Therapy vs. Western Medicine + Conventional Therapy

The results of 1 SR showed that XNJ can decrease the CSS score compared to low molecular dextran (D-40) (1 RCT, WMD = 0.63, 95% CI −0.83 to 2.09, *p* = 0.40) (Xu et al., 2005). While 1 SR showed a similar result, it pooled two RCTs comparing XNJ to D-40 and Cerebrolysin, respectively (2 RCTs, WMD = −4.48, *p* = 0.67, 95% CI −5.19 to −3.78) (Wang et al., 2006). There were no improvements in neurological function when compared with Citicoline and Venoruton, respectively (1 RCT, WMD = −2.3, *p* = 0.34, 95% CI −2.42 to 7.02; 2 RCTs, WMD = 7.05, 95% CI −6.46 to 7.65, *p* < 0.00,001, *I* ^*2*^ = 98.8%) (Li et al., 2013).

##### Scale 3: European Stroke Scale

Only 1 SR assessed the neurologic deficit score with ESS; the result combined four different interventions in the control group (Danshen, danhong, citicoline, and edaravone); this means there was high heterogeneity (4 RCTs, WMD = −0.32, 95% CI −1.39 to 0.74, *p* < 0.00001, *I* ^*2*^ = 99.6%) (Li et al., 2013).

##### Outcome 2: Activity of Daily Living

The result of 1 SR indicated XNJ combined with CT could significantly improve the ADL of acute IS patients when compared to CT (5 RCTs, MD = 10.23, 95% CI 9.47 to 10.99, *p* < 0.00001, *I* ^*2*^ = 0%) (Ma et al., 2017).

##### Outcome 3: Consciousness

###### Scale: Glasgow coma scale

Three SRs assessed coma with the GCS (Lin et al., 2010; Ma et al., 2017; Liao et al., 2019), but the results were different. The results of 2 SRs showed there were no significant differences between the XNJ combined with CT group and the CT group (2 RCTs, MD = 1.00, 95% CI −0.96 to 2.96, *p* = 0.32,*I* ^*2*^ = 79%; 4 RCTs, SMD = 0.67, 95% CI −0.40 to 1.74, *p* = 0.22, *I* ^*2*^ = 96.5%) (Lin et al., 2010; Ma et al., 2017), while another SR indicated that XNJ with CT was more effective than CT by increasing the scores of GCS (4 RCTs, MD = 2.46, 95% CI 2.06 to 2.86, *p* < 0.0001, *I* ^*2*^ = 76% (Liao et al., 2019).

##### Outcome 4: Mortality

In total, 4 SRs mentioned mortality in the Methods section (Xu et al., 2005; Wang et al., 2006; Li et al., 2013; Ma et al., 2013), but 3 SRs finally reported mortality in the Results section (Xu et al., 2005; Wang et al., 2006; Li et al., 2013), and 1 SR pooled the data of IS and cerebral hemorrhage (Wang et al., 2006). Two SRs compared XNJ with Danshen injection and included the same RCTs (Xu et al., 2005; Li et al., 2013); the results showed XNJ might decrease the mortality (3 RCTs, RR = 0.26, 95% CI 0.07 to 1.01, *p* = 0.05, *I* ^*2*^ = 0; 3 RCTs, RR = 0.31, 95% CI 0.14 to 0.70, *p* = 0.005, *I* ^*2*^ = 0). One SR also compared XNJ with Cerebrolysin, and there were no statistical differences between the two groups (1 RCT, RR = 0.92, 95% CI 0.14 to 6.27) (Xu et al., 2005).

##### Outcome 5: Infarct Size

One SR showed that XNJ could reduce infarction size when compared with CT (2RCTs, MD = −1.83, 95% CI −2.49 to −1.16, *p* < 0.00001, *I* ^*2*^ = 78%) (Ma et al., 2017).

#### Surrogate Outcomes

##### Outcome 1: Serum Levels of Matrix Metalloproteinase-2 (MMP-2), MMP-9, and NO

One SR summarized the serum levels of MMP-2 and MMP-9 (Ma et al., 2017). Compared with the CT group, XNJ significantly reduced the serum levels of MMP-2 and MMP-9 (2 RCTs, MD = −11.24, 95% CI −20.83 to −1.65, *p* = 0.02, *I* ^*2*^ = 76%; 5 RCTs, MD = −25.08, 95% CI −35.49 to −14.67, *p* < 0.00001, *I* ^*2*^ = 66%). The results of another SR showed that XNJ could improve the serum level of NO when compared to CT (2 RCTs, SMD = 1.72, 95% CI 1.12 to 3.85, *p* = 0.0003) (Lin et al., 2010).

##### Outcome 2: Hemorheological Parameters

The results of hemorheological parameters mainly contained whole blood viscosity (WBV), plasma viscosity (PV), hematocrit (HCT), and fibrinogen (FIB). Two SRs indicated that when comparing to CT, XNJ was more effective in improving WBV (5 RCTs, MD = −1.44, 95% CI −2.18 to 0.70, *p* = 0.001, *I* ^*2*^ = 87%; 4 RCTs, MD = −0.80, 95% CI −1.44 to −0.16, *p* = 0.01, *I* ^*2*^ = 88%), PV (5 RCTs, MD = −0.22, 95% CI −0.37 to −0.07, *p* = 0.003, *I* ^*2*^ = 73%; seven RCTs, MD = −0.28, 95% CI −0.44 to −0.12, *p* = 0.0005, *I* ^*2*^ = 94%), and HCT (two SRs included the same RCTs; 5 RCTs, MD = −3.63, 95% CI −6.23 to −1.03, *p* = 0.006, *I* ^*2*^ = 96%) (Ma et al., 2017; Wang et al., 2017). Besides, the result of 1 SR showed XNJ also could reduce FIB (3 RCTs, MD = −1.14, 95% CI −1.70 to −0.57, *p <* 0.000 1, *I* ^*2*^ = 95%) (Wang et al., 2017).

##### Outcome 3: Hemodynamic Parameters and Blood Fat

The results from 1 SR indicated that compared with the CT (Ma et al., 2017), XNJ could remarkably increase the peak-flow rate and average velocity (2 RCTs, MD = 12.66, 95% CI 10.50 to 14.81, *p* < 0.00001, *I* ^*2*^ = 71%; 2 RCTs, MD = 9.90, 95% CI 8.63 to 11.17, *p* < 0.00001, *I* ^*2*^ = 81%). XNJ could significantly reduce the levels of cholesterol and triglyceride in blood (MD = −1.06, 95% CI −1.21 to −0.92, *p* < 0.00001, *I* ^*2*^ = 0; MD = −1.05, 95% CI −1.12 to −0.97, *p* < 0.00001, *I* ^*2*^ = 0).

##### Outcome 4: Endothelin

Two SRs reported the pooled results of endothelin (Wang et al., 2006; Lin et al., 2010). Compared to CT, XNJ could reduce the level of endothelin (2 RCTs, SMD = −0.50, 95% CI −0.81 – −0.19, *p* = 0.001) (Lin et al., 2010). When comparing with other herbal injections (mailuoning and Danshen), XNJ could also reduce the level of endothelin (2 RCTs, WMD = −45.14, 95% CI −52.81 to −37.46, *p* < 0.00001), but with no subgroup analysis (Wang et al., 2006).

#### Composite Outcomes

##### Outcome: Total Effective Rate

Nine out of 10 included SRs defined total effective rate as a primary outcome (Xu et al., 2005; Li, 2006; Wang et al., 2006; Lin et al., 2010; Li et al., 2013; Ma et al., 2013; Chen and Gu, 2017; Ma et al., 2017; Wang et al., 2017). The total effective rate was a compound outcome and total effective rate = (basically cured patients + markedly improved patients + improved patients)/total number of patients. “Basically cured” meant CSS decreased 91–100%, “markedly improved” represented CSS decreased 46–90%, “improved” represented CSS decreased 18–45%, while 0–17% meant invalid and 0 or less meant deterioration (The Fourth National Academic Conference on Cerebro-vascular Diseases, 1997).

##### Xingnaojing Injection + Conventional Therapy vs. Conventional Therapy

Five SRs reported that XNJ could improve total effective rate with no heterogeneity (Lin et al., 2010; Li et al., 2013; Chen and Gu, 2017; Ma et al., 2017; Wang et al., 2017) (38 RCTs, *I* ^*2*^ = 0, OR = 3.56, 95% CI 2.94 to 4.32, *p* < 0.00001; 23 RCTs, *I* ^*2*^ = 0, RR = 1.22, 95% CI 1.18 to 1.27, *p* < 0.00001; 2 RCTs, *I* ^*2*^ = 0, RR = 1.30, 95% CI 1.11 to 1.53, *p* < 0.01; 21 RCTs, *I* ^*2*^ = 0, RR = 3.85, 95% CI 2.97 to 5.00, *p* < 0.00001; 14 RCTs, *I* ^*2*^ = 0, OR = 3.70, 95% CI 2.64 to 5.17, *p* < 0.00001), while another 2 SRs showed the opposite result (Xu et al., 2005; Li, 2006) (1 RCT, RR = 0.94, 95% CI 0.72 to 1.24; 3 RCTs, *I* ^*2*^ = 0, RR = 1.04, 95%CI 0.88–1.23).

##### Xingnaojing Injection + Conventional Therapy vs. Other Therapy + Conventional Therapy

The results of 2 SRs showed XNJ could improve the total effective rate (Xu et al., 2005; Li et al., 2013) when compared to Danshen (7 RCTs, *I* ^*2*^ = 0, RR = 1.37, 95% CI 1.26 to 1.50; 4 RCTs, *I* ^*2*^ = 48.1%, RR = 1.26, 95% CI 1.12 to 1.42, *p* = 0.0002), Citicoline (3 RCTs, *I* ^*2*^ = 0, RR = 1.37, 95% CI 1.20 to 1.57, *p* < 0.01; 1 RCT, RR = 1.30, 95% CI 1.03–1.63), and Mailuoning (1 RCT, RR = 1.43, 95% CI 1.09 to 1.86, *p* < 0.01; 1 RCT, RR = 1.36, 95% CI 1.06 to 1.75, *p* < 0.01). But there was no statistical difference when compared to Cerebrolysin (the same RCT, RR = 0.99, 95% CI 0.84 to 1.16, *p* > 0.05). One of the SRs also showed positive result when compared to D-40 (1 RCT, RR = 1.23, 95% CI 1.02, 1.47, *p* < 0.05) and edaravone (1 RCT, RR = 1.50, 95% CI 1.09 to 2.06, *p* = 0.01). But there were no statistical difference between the two groups when compared to Hetastarch (2 RCTs, RR = 0.97, 95% CI 0.80 to 1.17, *p* > 0.05) and Venoruton (1 RCT, RR = 1.16, 95% CI 0.97 to 1.38, *p* > 0.05) (Li et al., 2013). Another SR showed positive result when compared to Dextran-40 (1 RCT, RR = 1.44, 95% CI 1.24–1.60) (Xu et al., 2005). One SR pooled the data of all other therapies in the control group with no subgroup analysis (Wang et al., 2006), there was no heterogeneity (*p* = 0.28), and the result showed XNJ could improve total effective rate (8 RCTs, OR = 2.75, 95% CI 1.90–3.99).

There was still one SR that considered the quality of included RCT as a heterogeneity factor but with no limitations on treatment in the control group (Ma et al., 2013). The results of the two subgroups showed XNJ could improve the total effective rate (JADAD = 2, 2 RCTs, *I* ^*2*^ = 0, RR = 1.08, 95% CI 0.93 to 1.25; JADAD = 1, 7 RCTs, *I* ^*2*^ = 0, RR = 1.36, 95% CI 1.20–1.55).

### Safety of Xingnaojing Injection for Acute Ischemic Stroke

A total of 8 SRs mentioned adverse events (Xu et al., 2005; Li, 2006; Wang et al., 2006; Lin et al., 2010; Li et al., 2013; Ma et al., 2013; Wang et al., 2017; Liao et al., 2019), among which 3 SRs reported that there were no adverse events in the included original RCTs (Li, 2006; Lin et al., 2010; Ma et al., 2013) and 5 SRs reported adverse events (Xu et al., 2005; Wang et al., 2006; Li et al., 2013; Wang et al., 2017; Liao et al., 2019). The patient numbers of adverse events in the treatment group and the control group were 17 *VS* 21 (Wang et al., 2017), 2/0 (Xu et al., 2005; Li et al., 2013), 4/0 (Wang et al., 2006), and 8/3 (Liao et al., 2019), respectively. The adverse events in the XNJ group included slight skin rashes, nausea or vomiting, headache, dizziness, slight drop in blood pressure, and gastrointestinal reactions, while the CT group in 1 SR reported adverse events such as slight skin rashes, nausea or vomiting, headache, dizziness, drop in blood pressure, and gastrointestinal reactions (Wang et al., 2017). And the symptoms were improved by slowing down the drip rate of infusion and treating symptomatically; all the patients completed the trials. The remaining 2 SRs did not mention adverse events (Chen and Gu, 2017; Ma et al., 2017). The included SRs indicated that the adverse events in the CT group were less than that in the XNJ combined CT group, but there was no statistical difference between the two groups.

### Quality Assessment

#### Methodological Quality of Systematic Reviews

The results of AMSTAR 2 assessment are shown in Table 2. The overall quality of 10 SRs (100%) were rated as critically low (Xu et al., 2005; Li, 2006; Wang et al., 2006; Lin et al., 2010; Li et al., 2013; Ma et al., 2013; Chen and Gu, 2017; Ma et al., 2017; Wang et al., 2017; Liao et al., 2019), as the critical items were poorly reported. Only 1 SR (10%) reported 50% of the 16 items (Wang et al., 2017), while the remaining 9 SRs (90%) reported just less than half of the items on AMSTAR 2 (Xu et al., 2005; Li, 2006; Wang et al., 2006; Lin et al., 2010; Li et al., 2013; Ma et al., 2013; Chen and Gu, 2017; Ma et al., 2017; Liao et al., 2019). For the critical items, no SRs reported either predefined protocol (item 2) or comprehensive search strategy (item 4) completely. Just 1 SR (10%) provided the list of excluded studies and gave the reasons for exclusion (item 7) (Ma et al., 2017). When it comes to assessment of risk of bias (RoB), 3 SRs (30%) considered random sequence allocation and selection of the outcome report (item 9) (Ma et al., 2017; Wang et al., 2017; Liao et al., 2019), the other 7 SRs only assessed the unconcealed allocation and blinding (Xu et al., 2005; Li, 2006; Wang et al., 2006; Lin et al., 2010; Li et al., 2013; Ma et al., 2013; Chen and Gu, 2017), while only 1 SR (10%) accounted for RoB in individual studies when interpreting the results (item 13) (Wang et al., 2017). As for statistical combination, 6 SRs (60%) combined the result with appropriate methods (item 11) (Xu et al., 2005; Li et al., 2013; Ma et al., 2013; Ma et al., 2017; Wang et al., 2017; Liao et al., 2019). The last critical item (item 15) was relevant to publication bias, 5 SRs (50%) reported it completely (Wang et al., 2006; Lin et al., 2010; Chen and Gu, 2017; Wang et al., 2017; Liao et al., 2019). For the nine noncritical items, no SRs gave reasons for including only RCTs (item 3) or reported the included SRs in adequate detail, especially the timeframe for follow-up (item 8). All SRs did not report or try to find the sources of funding for individual RCTs included in the review (item 10), nor did they assess the potential impact of RoB in individual RCTs (item 12). The most well-reported (70%) item was the components of patient, intervention, control group, and outcome (item 1) (Xu et al., 2005; Wang et al., 2006; Lin et al., 2010; Li et al., 2013; Ma et al., 2017; Wang et al., 2017; Liao et al., 2019), 6 SRs (60%) mentioned they conducted study selection (item 5) and data extraction (item 6) using two reviewers (Xu et al., 2005; Wang et al., 2006; Lin et al., 2010; Ma et al., 2017; Wang et al., 2017; Liao et al., 2019). Besides, 5 SRs (50%) explored the possible reasons for the heterogeneity and discussed the effect on the results caused by the heterogeneity (item 14) (Xu et al., 2005; Li et al., 2013; Ma et al., 2017; Wang et al., 2017; Liao et al., 2019). And 2 SRs (20%) claimed there was no conflict of interest in the review (item 16) (Ma et al., 2013; Ma et al., 2017).

**TABLE 2:** Methodological quality assessment by AMSTAR 2.

Included SRs	Item 1	Item 2	Item 3	Item 4	Item 5	Item 6	Item 7	Item 8	Item 9	Item 10	Item 11	Item 12	Item 13	Item 14	Item 15	Item 16	Total yes	Overall quality
Li. (2006)	N	PY	N	PY	N	N	N	PY	PY	N	N	N	N	N	N	N	0	Critically low
Ma et al. (2017)	Y	PY	N	N	Y	Y	Y	PY	Y	N	Y	N	N	N	N	Y	7 (43.75%)	Critically low
Ma et al. (2013)	N	N	N	N	N	N	N	N	N	N	Y	N	N	Y	N	Y	3 (18.75%)	Critically low
Wang et al. (2017)	Y	PY	N	PY	Y	Y	N	PY	Y	N	Y	N	Y	Y	Y	N	8 (50%)	Critically low
Li et al. (2013)	Y	PY	N	PY	N	N	N	PY	PY	N	Y	N	N	Y	N	N	3 (18.75%)	Critically low
Lin et al. (2010)	Y	PY	N	PY	Y	Y	N	PY	PY	N	N	N	N	N	Y	N	4 (25%)	Critically low
Xu et al. (2005)	Y	PY	N	PY	Y	Y	N	PY	PY	N	Y	N	N	Y	N	N	5 (31.25%)	Critically low
Chen and Gu, (2017)	N	PY	N	PY	N	N	N	PY	PY	N	N	N	N	N	Y	N	1 (6.25%)	Critically low
Wang et al. (2006)	Y	PY	N	PY	Y	Y	N	PY	PY	N	N	N	N	N	Y	N	4 (25%)	Critically low
Liao et al. (2019)	Y	PY	N	PY	Y	Y	N	PY	Y	N	Y	N	N	Y	Y	N	7 (43.75%)	Critically low
In total of “Y”	70%	0	0	0	60%	60%	10%	0	30%	0	60%	0	10%	50%	50%	20%		

SRs, systematic reviews; Y, yes; PY, partial yes; N, no.

#### Evidence Quality of Outcomes

The important clinical outcomes were assessed except for those in the descriptive analysis. The results of evidence quality rated by GRADE are shown in Table 3.

**TABLE 3:** Quality of evidence in included SRs with GRADE.

Included SRs	Outcome	No. of RCT (patient intervention/control group)	Certainty assessment					Certainty
			Risk of bias	Inconsistency	Indirectness	Imprecision	Publication bias	
Ma et al. (2017)	NIHSS	12 (632/641) 18 (816/809)	Serious^a^ Serious^a^	Serious^b^ Serious^b^	Not serious Not serious	Serious^c^ Serious^c^	Undetected Undetected	Very low Very low
	CSS							
	ADL	5 (214/212)	Serious^a^	Not serious	Not serious	Serious^c^	Undetected	Low
	GCS	2 (72/68)	Serious^a^	Serious^b^	Not serious	Serious^c^	Undetected	Very low
	Infarct size	2 (99/99)	Serious^a^	Not serious	Not serious	Serious^c^	Undetected	Low
Li et al. (2013)	Mortality	3 (119/118)	Not serious	Not serious	Not serious	Serious^c^	Undetected	Moderate
	NIHSS	3 (212/217)	Serious^a^	Not serious	Not serious	Serious^c^	Undetected	Low
	ESS	4 (217/211)	Serious^a^	Serious^b^	Not serious	Serious^c^	Undetected	Very low
	CSS	6 (295/274)	Serious^a^	Serious^b^	Not serious	Serious^c^	Undetected	Very low
	CSS	1 (48/49)	Serious^a^	Undetected	Not serious	Serious^c^	Undetected	Low
	CSS	1 (32/28)	Serious^a^	Undetected	Not serious	Serious^c^	Undetected	Low
	CSS	2 (63/60)	Serious^a^	Serious^b^	Not serious	Serious^c^	Undetected	Very low
	CSS	1 (27/26)	Serious^a^	Undetected	Not serious	Serious^c^	Undetected	Low
Lin et al. (2010)	GCS	4 (236/223)	Serious^a^	Serious^b^	Not serious	Serious^c^	Undetected	Very low
Xu et al. (2005)	Mortality	3 (119/108)	Not serious	Not serious	Not serious	Serious^c^	Undetected	Moderate
	Mortality	1 (50/46)	Not serious	Undetected	Not serious	Serious^c^	Undetected	Moderate
Chen and Gu, (2017)	CSS	6 (300/294)	Serious^a^	Serious^b^	Not serious	Serious^c^	Undetected	Very low
Wang et al. (2006)	CSS	2 (168/158)	Serious^a^	Serious^b^	Not serious	Serious^c^	Undetected	Very low
Liao et al. (2019)	GCS	4 (268/265)	Serious^a^	Serious^b^	Not serious	Serious^c^	Undetected	Very low

a Hiding or blinding was not used.

b The difference of point effect size between studies was large, confidence interval overlap between studies was little, the heterogeneity test was significant, or the I^2^ was large.

c Small number of events, or confidence interval was too wide.

There were 20 important outcomes in the 10 SRs, 7 (35%) outcomes with low-quality evidence, 10 (50%) with very low–quality evidence, and 3 (15%) with moderate-quality evidence. Imprecision (100%) was the most common reason for downgrading the quality of evidence due to the small number of events or a wide confidence interval. Risk of bias ranked second (85%) with 17 outcomes, except for three outcomes of mortality in 2 SRs (Xu et al., (005)2, Li et al., 2013), followed by inconsistency for 10 outcomes (50%). No evidence was downgraded because of indirectness. Publication bias was not detected.

## Discussion

### Summary of Findings

In recent years, more and more clinical studies and SRs about the effectiveness of XNJ in acute IS were published between 2005 and 2019. The purpose of this overview of SRs was to provide an overall evaluation and summary of the current evidence about the effectiveness of XNJ on acute IS. Of the 10 SRs and MAs identified (Xu et al., 2005; Li, 2006; Wang et al., 2006; Lin et al., 2010; Li et al., 2013; Ma et al., 2013; Chen and Gu, 2017; Ma et al., 2017; Wang et al., 2017; Liao et al., 2019), the methodological quality were critically low assessed by AMSTAR2, especially for the poorly reported critical items including predefined protocol, comprehensive search strategy, list of excluded studies, and reasons for exclusion. Besides, the quality of included original RCTs also influenced the quality of SRs. These 10 included SRs indicated that XNJ were used in combination with CT in the treatment of acute IS in most of the original RCTs. Very low– to low-quality evidence showed that XNJ combined with CT can improve the neurological deficits score no matter which scale is used. And moderate-quality evidence suggested that XNJ combined with CT could reduce mortality compared to Danshen injection. Both the XNJ and CT groups reported slight adverse events.

As for acute IS, no SRs reported the severity of acute IS and only 4 SRs reported the course of disease (Li, 2006; Wang et al., 2006; Ma et al., 2013; Wang et al., 2017). It remains unknown whether it is more beneficial to use XNJ for acute IS as soon as possible. A registry study (register number: NCT04275349), considering different timepoints of XNJ as the exposure group is being conducted to answer this question. We searched the ongoing registered trials thoroughly for trials on XNJ for acute IS that were registered on ClinicalTrials.gov in October 2020. Two multicenter RCTs (XNJ as an intervention group) are currently enrolling acute IS patients within 24 h of symptom onset, and the sample size ranges from 720 to 1,200 patients, which indicates that these data may be able to provide answers for some clinical questions.

When it comes to outcomes, neurological deficits, consciousness, and total effective rate were the frequently used outcomes in the included SRs. Three kinds of scales were used to assess neurological deficits, including NIHSS, ESS, and CSS. NIHSS is more widely used with good validity and reliability, while ESS has a high structural validity but needs to be further verified in clinical trials (Herndon, 2006). CSS is usually used in China, and both validity and reliability of CSS also need further verification. Conventionally, neurological function can only partially explain the health of the body and cannot be used as an outcome indicator alone; the clinical benefit of acute stroke patients is usually measured using the modified Rankin scale (mRS) (Broderick et al., 2017; Powers, 2020), but all the included SRs or MAs did not measure this outcome. GCS is used to assess the degree of coma, but it is not appropriate for stroke patients who are unconscious (Powers, 2020). Three SRs included in this overview showed controversial results (Lin et al., 2010; Ma et al., 2017; Liao et al., 2019). One included unconscious stroke patients and indicated that XNJ could improve the consciousness of patients (Liao et al., 2019), while the other 2 SRs showed no statistical difference and the consciousness condition of the included patients was not clearly reported. As we have mentioned above, the total effective rate is a compound outcome and mainly measured using CSS when evaluating the effectiveness on neurological deficits. Total effective rate combine 3 levels of CSS decrease (91–100%, 46–90% and 18–45%), and may exaggerate the efficacy and make type I errors in statistics (Mccoy, 2018; Yan et al., 2019; Zhang et al., 2020).

### Strengths and Limitations

This overview evaluated and summarized the current evidence about the effectiveness of XNJ on acute IS, classified the results according to different types of outcomes, and assessed the quality of evidence for clinical outcomes with GRADE. Although we conducted this overview according to the Cochrane handbook of overviews of reviews (Higgins Jpt, 2019), it still has some limitations. We focused on different timepoints for XNJ in acute IS but could not pool the data of the included SRs that were searched as this information in the SRs or original RCTs was not completely reported.

### Implications

Whether it is an SR or RCT to evaluate intervention effectiveness for acute IS in the future, researchers should consider measuring neurological deficits using a recognized and validated NIHSS and combine the robust endpoint outcomes including mortality and mRS. Besides, there are no specific outcomes for evaluating TCM treatment in acute IS; studies focused on core outcome measures for TCM treatment in acute IS have been registered on the website of Core Outcome Measures in Effectiveness Trials (COMET), which could provide a reference for the selection of core outcomes for acute IS with TCM treatment in the future.

For the methodological quality of SR, future SRs should register the protocol before commencing the study; more and more journals also require manuscript of SRs to provide the register protocol number. In addition, lists of excluded studies and reasons for exclusion, publication bias and RoB, and the influence of RoB in individual studies when present in the results should also be reported completely.

## Conclusion

Very low to low quality of evidence indicated XNJ combined CT could improve the neurological deficits of acute IS, but it remains unknown whether it is more beneficial to use XNJ for acute IS as soon as possible after the symptom onset. Well-designed large-scale RCTs with measurable validated endpoints are still needed in the future studies. Slight adverse events in the CT group were less than those in the XNJ combined CT group, and there were no serious adverse events reported, indicating that XNJ is relatively safe.

## Appendix 1

### Searching Strategies on PubMed:

#1. systematic review [mesh]

#2. review [tiab]

#3. meta-analysis [mesh]

#4. systematic review [tiab]

#5. meta-analysis [tiab]

#6. meta analysis [tiab]

#7. data pooling [tiab]

#8. data poolings [tiab]

#9. Overview, Clinical Trial [tiab]

#10. Clinical Trial Overview [tiab]

#11. Xingnaojing [tiab]

#12. 1 or 2 or 3 or 4 or 5 or 6 or 7 or 8 or 9 or 10

#13. 11 and 12

### Searching Strategies on CNKI:

SU=('中风'+'卒中'+'脑梗'+'脑栓塞'+'腔梗'+'脑血管病'+'脑缺血'+'脑出血') AND SU=醒脑静 AND SU=('系统综述'+'系统评价'+'系统'+'Meta分析'+'荟萃分析'+'汇总分析'+'集成分析'+'二次分析'+'衍生分析')
